# Model Informed Precision Dosing of Tacrolimus in Children Following Heart Transplant

**DOI:** 10.1002/jcph.70122

**Published:** 2025-10-19

**Authors:** Jadon S. Wagstaff, Shaun S. Kumar, Kimberly M. Molina, Joseph E. Rower

**Affiliations:** ^1^ Division of Clinical Pharmacology, Department of Pediatrics University of Utah School of Medicine Salt Lake City Utah USA; ^2^ Primary Children's Medical Center Intermountain Healthcare Salt Lake City Utah USA; ^3^ Division of Cardiology, Department of Pediatrics University of Utah School of Medicine Salt Lake City Utah USA; ^4^ Department of Pharmacology and Toxicology University of Utah College of Pharmacy Salt Lake City Utah USA; ^5^ Center for Human Toxicology University of Utah College of Pharmacy Salt Lake City Utah USA

**Keywords:** cardiac surgery, decision support tool, pediatric surgery, pharmacology, tacrolimus

## Abstract

Tacrolimus is a first‐line immunosuppressant used after solid organ transplantation that suffers from extensive intra‐ and inter‐patient variability and a narrow therapeutic window. Its critical role in a fragile population, coupled with the difficulties identifying and maintaining an appropriate dose within a given patient, make it an ideal candidate for population pharmacokinetic (popPK)‐guided individualized dosing approaches (i.e., model informed precision dosing, MIPD). We previously published a tacrolimus popPK model in pediatric heart transplant recipients that showed promise in its ability to predict future concentrations within an individual. Using that model, we developed a Bayesian forecasting decision support tool (DST) clinical use to more rapidly attain appropriate tacrolimus dosing in this population. After rigorous in silico testing of the DST's mathematical fidelity to the popPK model, we implemented the DST within a clinical trial (NCT04380311). Fifteen children between 6 months and 17 years of age had their tacrolimus doses guided by the DST to determine the time to stable therapeutic tacrolimus dosing (defined by three consecutive concentrations within the targeted therapeutic range). DST‐guided dosing achieved stable tacrolimus dosing ∼3 days faster (6.9 days, *P* = .03) as compared to a historical cohort (9.8 days). This was despite the poor performance of the DST in two children treated with continuous renal replacement therapy. These results demonstrate the clinical utility and benefit of the described DST, which is the first targeted to the pediatric heart transplant population. Rapid attainment of stable therapeutic tacrolimus dosing has benefits for the patient, clinician, and the healthcare system.

## Introduction

Heart transplantation is an accepted therapeutic option for children with congenital heart disease and cardiomyopathy.^1^ Nearly 500 pediatric heart transplants were performed in 2022 across the United States with improving outcomes in recent decades, though mortality from rejection, infection, and coronary vasculopathy remain significant.^2^, ^3^ Transplant survival in excess of 20 years following heart transplantation has been observed, with more than 70% of transplants expected to achieve greater than 5 year survival.^2^, ^4^ Much of this success can be attributed to the use of immunosuppressive therapy to prevent the rejection of the transplanted cardiac tissue.

**Article Summary**

**What is known**:

1. Tacrolimus is a primary immunosuppressant agent in preventing graft rejection in children receiving heart transplant.
2. Tacrolimus suffers from substantial inter‐ and intra‐patient pharmacologic variability, complicating dosing strategies.

**What this study adds**:

1. A previously described population pharmacokinetic model was used to build a decision support tool.
2. The decision support tool can be used to guide dosing that more rapidly yields therapeutic concentrations of tacrolimus following pediatric heart transplant.

The calcineurin inhibitors (CNI) tacrolimus and cyclosporine play a vital role in immunosuppressive therapy. Currently, tacrolimus is preferred in comparison to cyclosporine, due to its improved safety profile, especially with regard to hypertension and dyslipidemia.^5^, ^6^ Despite these advantages, tacrolimus is characterized as a drug with a narrow therapeutic window and exhibits extensive inter‐ and intra‐patient pharmacokinetic (PK) variability, which necessitates frequent drug monitoring to guide therapeutic dosing.^7^, ^8^, ^9^, ^10^, ^11^, ^12^, ^13^, ^14^, ^15^, ^16^ Unfortunately, the extensive PK variability complicates the interpretation of drug monitoring results. Indeed, a retrospective study comparing drug monitoring data against the resulting dose modification in our institution showed that approximately 50% of dose modifications do not follow the expected pattern (i.e., sub‐therapeutic concentrations are not followed by the expected dose increase).^17^ While this study did not characterize reasons for not following the expected pattern, some of which may be rational and valid, the finding nonetheless argues for objective approaches for guiding tacrolimus dosing to ensure optimal patient care. Furthermore, we found that approximately 40% of drug monitoring concentrations are outside the therapeutic target range for tacrolimus in heart transplant.^18^ These findings advocate the need for a model‐based decision support tool (DST, also known as model informed precision dosing in the literature) that can be used to guide tacrolimus dosing.

We previously described a population PK (popPK) model that identified the covariates age, renal function, and concomitant fluconazole use as significant contributors to variability in tacrolimus PK in pediatric heart transplant recipients.^18^ Additionally, we found that the model could be used to successfully predict future tacrolimus concentrations when as few as three concentrations were used to determine individualized estimates of PK parameters.^18^, ^19^ We utilized this model to build a clinician‐facing DST that can be used to guide tacrolimus dosing that achieves therapeutic concentrations during the inpatient stay period immediately post‐transplant. This study evaluates the ability and utility of the DST to accurately predict a safe and therapeutic tacrolimus dose with both in silico and in vivo approaches.

## Methods

### Data Collection

Study approval was granted by the University of Utah, Intermountain Health, and Primary Children's Hospital Institutional Review Board (IRB_00086042; IRB_00109297; and IRB_00124800). All study procedures followed the ethical guidelines outlined in the Declaration of Helsinki.

Data used to build the previously described population PK model^18^ and conduct *in silico* testing of the DST, including event times, dose amount, tacrolimus concentrations, and demographics, were collected retrospectively from the Intermountain Health Enterprise Data Warehouse. The retrospective study (i.e., historical cohort) included children receiving tacrolimus during an inpatient stay at Primary Children's Hospital in Salt Lake City, Utah within the first 6 weeks following heart transplant between the years of 2007 to 2013.^18^ Furthermore, children meeting these criteria must have had at least one dose of tacrolimus and one tacrolimus concentration measurement to be incorporated into the study. Clinical dosing information was verified by scanning a barcode on the patient's bracelet immediately prior to tacrolimus administration. Tacrolimus was typically administered twice daily, either orally or enterally through a nasogastric or nasojejunal tube. In addition to tacrolimus, all patients received mycophenolate as part of their immunosuppressive regimen, and milrinone was used to provide cardiac support post‐transplant.

Participants for the prospective clinical testing of the DST (IRB_124800, NCT04380311) were recruited and enrolled in the study before collection of their third sample for therapeutic drug monitoring (TDM) between 2020 and 2023. Parental permission was obtained for all participants, and assent was obtained from participants greater than 7 years of age. Eligibility criteria included being between the ages of 6 months and 17 years of age and the receipt of a heart transplant due to diagnosis of a congenital heart malformation or cardiomyopathy. Participants were excluded if they had been diagnosed with a significant comorbidity that would prevent study completion.

For both participant cohorts, tacrolimus TDM samples were collected following standard of care, typically a once‐daily trough concentration until discharge. Tacrolimus concentrations were determined from whole blood using a validated liquid chromatography‐tandem mass spectrometry (LC‐MS/MS) method at ARUP Laboratories. The assay was linear between 1 and 40 ng/mL. Sample times were determined relative to the first dose of tacrolimus.

### Pharmacokinetic Modeling

Constructing the popPK model used as a foundation for the DST has been described previously.^18^ Briefly, PK modeling utilized NONMEM software (v7.3, ICON Development Software, Ellicott City, MD) interfaced with PDx‐Pop (v5.0). The first order conditional estimation with interaction (FOCE‐I) method was used throughout model building and evaluation. Model selection was based on parsimony, objective function value (OFV), and visual diagnostic plots. The model was parameterized on elimination rate (k_e_), volume of distribution (V), and a fixed absorption rate (k_a_ = 3.43/h). Residual error used an additive model and between subject variability was included on k_e_ and V. Age was incorporated as a covariate on V, while both creatinine clearance and fluconazole use were included as covariates on k_e_.

### Building the DST

The DST was coded in client‐side JavaScript which interfaces with an HTML5 document. Client‐side JavaScript was chosen so that no personal health information is transmitted to any unauthorized servers. Patient covariates, prior tacrolimus doses and concentrations, and target tacrolimus parameters are input by the user via HTML5 form elements and/or uploaded from a CSV file. The JavaScript code captures the user input and, in conjunction with the popPK model, estimates an appropriate dose. The dose suggestion is adjusted to the individual through Bayesian optimization of the model parameters k_e_ and V. To adjust PK parameters to fit an individual, between subject variability parameters (ETA values) are optimized through coordinate descent minimization of an objective function to establish a maximum a posteriori probability (MAP) estimate.^20^, ^21^ Dose suggestions and a predicted concentration–time curve are displayed as an output. Code for the DST is provided in the Supplemental Information and is available on GitHub at https://github.com/jadonwagstaff/tacrodose_pht.

### In Silico Testing of the DST

We have previously demonstrated the popPK model's ability to predict an individual's future tacrolimus concentrations using as few as three observed concentrations for the individual.^18^ The concentration‐time data from the 30 retrospective patients collected from 2007 to 2013 shown in Table 1 was used for in silico testing of the DST. These patients had between 2 and 50 doses and 1 to 21 trough concentration measurements with a total of 935 doses and 395 trough concentration measurements. These data were used to test the mathematical fidelity of the DST to the previously published popPK model, assess the DST's ability to provide accurate dosing recommendations, and confirm that it accurately estimates ETA values. This is accomplished through three tests, described below.

**Table 1:** Demographic Characteristics of the Retrospective Study Population Used to Build the Population PK Model^18^ and the Prospective Study Population Used to Clinically Test the DST.

	Retrospective Cohort	Prospective Cohort
Study period	1/2007‐12/2013	7/2020‐6/2023
Subjects	30	15
Sex		
Male	19	7
Female	11	8
Race		
Caucasian	28	13
African‐American	1	2
Other	1	0
Fluconazole use		
Yes	15	3
No	15	12
Age (year)		
Median (range)	5.7 (0.1, 17.7)	11.0 (1.10, 17.3)
Weight (kg)		
Median (range)	28.9 (7.0, 77.2)	35.8 (8.30, 90.8)
Creatinine clearance (mL/min/1.73 m^2^)		
Median (range)	122.4 (15.6, 442.2)	112.9 (55.9,181.5)
Transplant indications		
Congenital heart disease	14	10
Cardiomyopathy	16	5
Arrhythmia	0	0
Dose (mg/kg/day)		
Median (range)	0.09 (0.020, 0.490)	0.07 (0.04,0.49)

Mathematical fidelity of the DST to the popPK model was interrogated by comparing predicted model parameters and concentrations to output from NONMEM. Given the same dosing time and amount, covariates, and concentration measurement times for each of the 30 patients, 10 sets of simulated ETA values per patient were used to simulate 300 patient concentration–time datasets. The tacrolimus trough concentrations and individual k_e_ and V model parameters were predicted for each simulated patient by NONMEM using the popPK model. The dosing, covariate, and simulated ETA values were also input into the DST to predict model parameters and concentrations, then the differences between these predictions and NONMEM predictions were evaluated.

The DST dose suggestion was verified by using the 300 previously described patient covariates and simulated ETA values to predict a 12 h oral tacrolimus dose that targets a 12 ng/mL trough concentration. Using the predicted dose and maintaining the last‐observed patient covariates, 200 oral doses in 12 h increments were input into the DST. Trough concentrations after 200 doses were predicted for each simulated patient and compared to the 12 ng/mL target.

Lastly, the DST ETA value estimation, and intrinsically, the DST objective function minimization algorithm were evaluated. Dosing times and amounts, observed trough concentrations, and covariate information from the 30 retrospective patients were input into both the DST and NONMEM and patient‐specific MAP ETA value estimates were compared. ETA values are log‐normally distributed, so ETA values estimated by the DST and NONMEM are on a log‐scale. Since ETA values are estimated on a log‐scale, ETA values are exponentiated before being multiplied by baseline k_e_ and V values to get patient‐specific concentration predictions. Because of this, ETA values estimated by NONMEM and the DST are exponentiated before comparison for a more accurate assessment of the differences in effect on k_e_ and V.

### Clinical Testing of the DST

After completing in silico testing, we conducted a prospective study (NCT04380311) to assess the clinical utility and benefit of the DST in pediatric heart transplant recipients. Target enrollment for this study was 16 children, to provide an a priori power of 99% to demonstrate a 4 day reduction in the time to stable tacrolimus dosing. Once a participant was identified and enrolled, study staff queried the necessary data from the participant's electronic medical record (EMR) to build the input dataset. The input dataset was then loaded into the DST and utilized to predict a dose for the participant that would yield a trough concentration within the therapeutic target (10 to 14 ng/mL).^22^, ^23^ The dose was then relayed to the clinical team (consisting of physicians, advanced practitioners, and clinical pharmacists) for implementation in the participant. The input dataset and dose guidance were updated after every collected blood sample until the participant achieved steady (i.e., three consecutive) concentrations within the therapeutic target range. The average time to steady therapeutic concentrations was compared between the DST and a historical cohort consisting of the patients used to construct the population PK model the DST is based on (9.8 days for 48 patients between 2007 and 2015) using a two‐sided t‐test (*P* < .05) in GraphPad Prism (San Diego, CA).

## Results

### Building the DST

**Figure 1:**
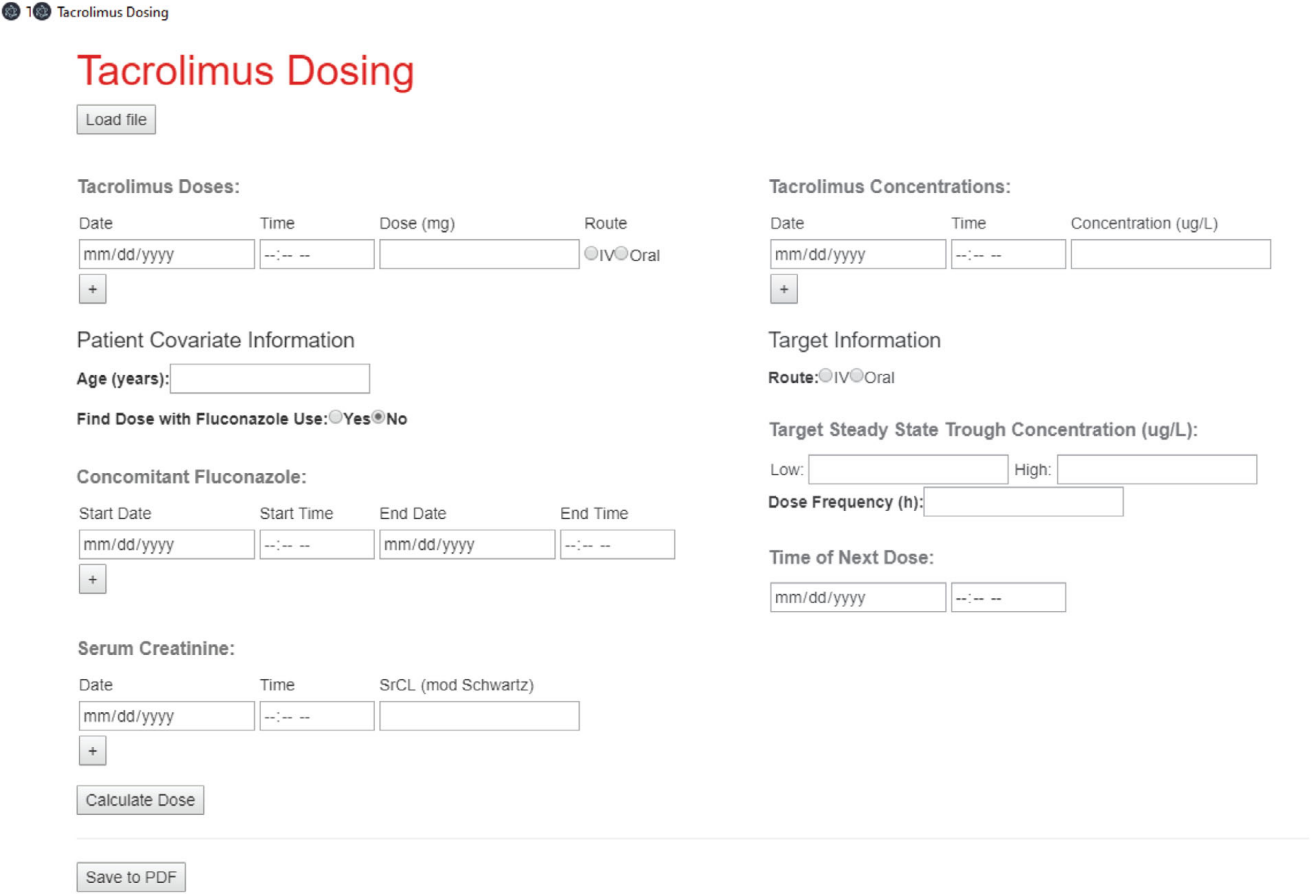
User interface for the DST, allowing the user to manually enter data or upload data from a properly formatted spreadsheet.

**Figure 2:**
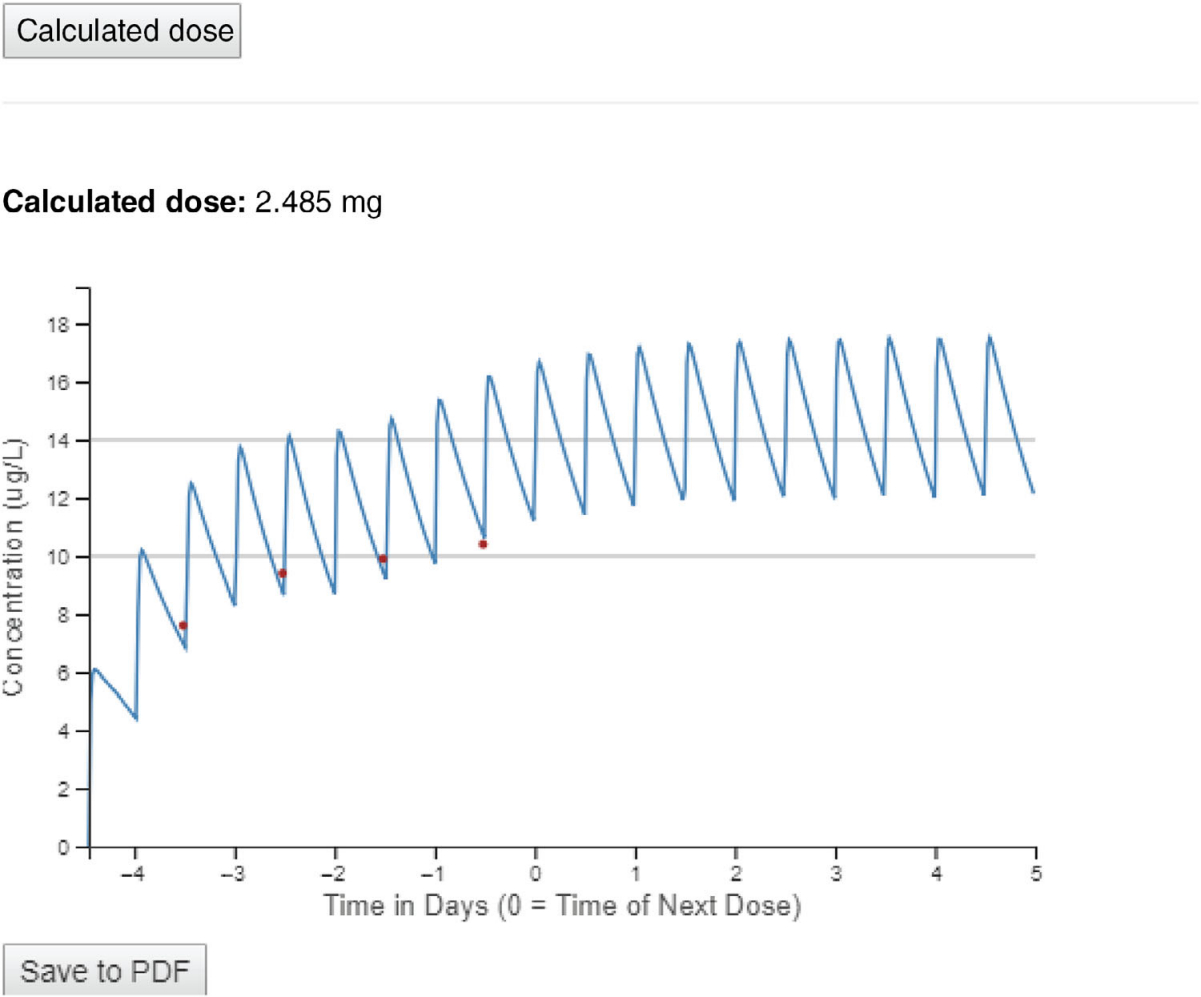
DST output, including a predicted dose and a plot of observed concentrations (points) and predicted concentrations (line). Predicted concentrations after day “0” represent concentrations that would occur as a result of the predicted dose.

The user interface for the DST is shown in Figure 1. In addition to manually entering data into appropriate sections of the DST, the user has the option to upload data from a .xls or .xlsx spreadsheet. After data is entered and the DST executed, the dose suggestion and a plot showing the observed concentration data with an overlaid model‐based concentration–time curve is shown (Figure 2). The user then has the option to save both the input and the output of the DST into a .pdf file for future reference. The DST is presently hosted locally, to avoid the potential risk for a breach of HIPAA‐protected information. Importantly, the run time for the tool is negligible—taking less than a second to run the code to verify the DST accuracy for 300 simulated patients (described in the next section).

### In Silico Testing of the DST

For the DST to accurately replicate the previously published PK model's ability to predict future concentrations and guide dosing, the DST must generate accurate PK parameters and concentration predictions given patient covariates and ETA values, the DST must give an accurate dose recommendation, and the DST must accurately estimate ETA values given observed patient covariates and trough concentrations. Results of the DST in silico testing are summarized in Table 2.

**Table 2:** In Silico Testing Results.

Parameter	Maximum Difference (%)	Mean Difference (%)
1. NONMEM simulation versus DST		
Elimination rate constant (k_e_, 1/h)	0.00997	0.00113
Volume of distribution (V, L)	0.00803	0.000919
Predicted concentration (ng/mL)	0.0155	0.00000848
2. DST dose suggestion (trough concentration target of 12 ng/mL)		
Difference between target and predicted (ng/mL)	0.0407	0.00511
3. NONMEM ETA estimates versus DST		
Exponentiated ETA value for k_e_	0.211	0.0636
Exponentiated ETA value for V	0.230	0.0528

The differences between NONMEM and DST calculations of predicted k_e_, V, and concentrations given dosing, covariate, and simulated ETA values were low with a maximum difference of 0.0155%. These differences are small enough that they may be attributed to rounding error. Given the high accuracy of predicted concentrations, the predicted trough concentration after long‐term dosing with the recommended tacrolimus dose should be close to the target of 12 ng/mL if the dose recommendation is correct. The trough concentrations after 200 recommended 12h doses given last‐observed patient covariates and simulated ETA values are all within 0.0407% of 12 ng/mL which verifies that the correct dose is being recommended. Dose recommendations that are fit to an individual patient are dependent on accurate ETA value estimates. Exponentiated patient ETA values estimated by the DST were within 0.230% of those estimated by NONMEM, demonstrating that the coordinate descent minimization of the objective function produces appropriate MAP ETA estimates.

### Clinical Testing of the DST

**Figure 3:**
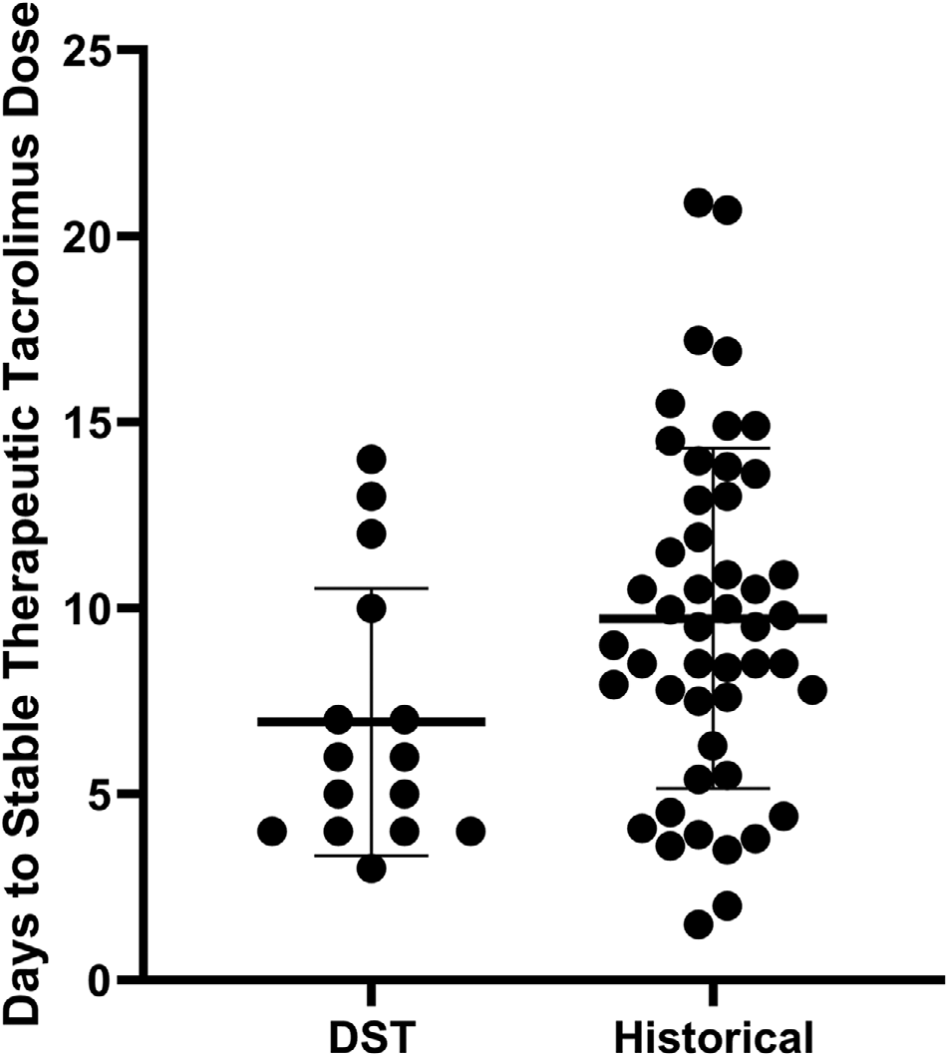
Comparison of historical time to stable therapeutic concentration (i.e., three consecutive concentrations in the target tacrolimus therapeutic range) versus the same value for participants with DST‐guided dosing.

A total of 17 participants were enrolled in the prospective trial testing the utility and benefit of the DST. Two participants were excluded from analysis, the first due to an inability to access the necessary data to build the input dataset from the patient's EMR and the second due to the participant's renal insufficiency limiting physician willingness to utilize the DST guidance. A total of 15 participants were used for the analysis (yielding a post hoc power of 80%), with demographics shown in Table 1. The time to stable therapeutic tacrolimus concentration (i.e., three consecutive concentrations between 10 and 14 ng/mL) in these 15 participants took an average (standard deviation, SD) of 6.93 (3.59) days, with a range between 3 and 14 days. Of the 15, 11 achieved stable therapeutic concentrations within 7 days of initiating tacrolimus dosing. Of the remaining four individuals, two received continuous renal replacement therapy (CRRT) and one was later found to possess the CYP3A5*1/*3 genotype, which has previously been associated with greater tacrolimus dose requirement.^24^, ^25^, ^26^ Clinical application of the DST was able to achieve stable tacrolimus concentrations approximately 3 days earlier than the historic cohort (Figure 3, *P* = .03).

## Discussion

The development and use of personalized pharmacotherapy tools such as the described DST have great potential to improve clinical outcomes, especially for drugs like tacrolimus with narrow therapeutic indices. The need for these tools is especially great in the pediatric population, where drug disposition and dose–response relationships are highly dynamic, yet less well studied than adults.^27^, ^28^, ^29^, ^30^, ^31^, ^32^ Moreover, in the transplant setting, where the patients in need of a transplant organ far outnumber the organs available for transplant,^33^, ^34^ it is critical that patients receive optimal immunosuppressive therapy to prevent organ rejection.^8^, ^35^, ^36^, ^37^ Immunosuppressant regimens require continual monitoring to ensure drug exposures that are effective, yet do not result in toxicity.

A recent systematic review describes available DST that provides model informed precision dosing (MIPD) of tacrolimus in solid organ transplantation.^38^ Of the 80 articles identified by Hoffert et al in this review, few (6.3%) described data from heart transplants, with the majority of studies focused on either kidney (51.3%) or liver (32.5%).^38^ Moreover, of the studies used to build DST, only six were built using pediatric data,^39^, ^40^, ^41^, ^42^, ^43^, ^44^ and none were built for use in pediatric heart transplant recipients. Pediatric heart transplant‐specific population PK models and thus DST for this population, remain a large gap in the existing literature describing the use tacrolimus in solid organ transplant. In fact, another recent systematic review by Khamlek et al found our prior work to be the only description of tacrolimus in a pediatric heart transplant population, and recommended future work to focus on this population.^45^ Notably, the elevated risk of rejection following heart transplant typically leads to higher tacrolimus target concentrations relative to liver or kidney transplantation, further narrowing the therapeutic window for these patients and increasing the need for model informed dosing decisions in this population. The absence of other work specific to the pediatric heart transplant field demonstrates the novelty and critical need for the described DST to support the clinical care of this population.

The constructed DST provides personalized dose guidance for pediatric heart transplant recipients. Doses are tailored to the individual based on age, renal function, and the concomitant use of fluconazole, a known CYP3A4/5 inhibitor.^18^, ^19^, ^46^, ^47^, ^48^, ^49^ Data can be input into the DST directly, or more conveniently, by loading an input dataset with the required data. Output data includes the predicted dose in addition to a plot which provides insight into the accuracy of the dose prediction. Finally, each prediction can be saved as a .pdf file to document the input and output data. *In silico* DST testing demonstrated high fidelity to the estimates generated within NONMEM. As NONMEM represents a gold standard software in the pharmacometrics world, the concordance of these estimates supports the appropriateness of the objective function minimization approach within the DST and the reliability of the dose predictions generated by the DST, which is a critical step in support of the clinical application of the DST.

Having demonstrated the *in silico* accuracy of the DST, we then evaluated the clinical application and benefits of the DST. The DST decreased the time to stable tacrolimus concentrations by ∼3 days relative to a historical cohort, indicating that the DST predicted the appropriate dose for study participants more rapidly than standard of care approaches. There are many potential benefits of more rapidly attaining appropriate tacrolimus dosing. First, is the reduced need for TDM once the appropriate dose is identified, decreasing the number of needlesticks endured by the patient. Additionally, identifying the correct dose may lead to discharging the patient home earlier. All of the above benefit the patient, the clinical team, and the entirety of the healthcare system. Next, rapid attainment of an optimal immunosuppressant dose may benefit patient outcomes. Spending more time within the therapeutic range early after transplantation can reduce the risk of rejection events (subtherapeutic dosing) or nephrotoxicity and opportunistic infection (supratherapeutic dosing). A final benefit to DST‐guided dosing is the ability to predict how dosing requirements will change when clinical status changes, which is particularly advantageous when a child stops prophylactic azole antifungal therapy. Such a change eliminates the azole‐mediated inhibition of CYP3A5,^46^, ^47^, ^48^, ^49^ resulting in a need for a compensatory increase of the administered tacrolimus dose.

Despite the potential benefits that the described DST can have for the pediatric heart transplant population, there are key limitations. First, the DST is currently hosted on a local computer, rather than being fully integrated into the EMR. For the DST to have a more widespread impact, both internal and external to our institution, it must be implementable from the EMR. While integrating this DST into the Cerner‐based EMR will likely be straightforward, it requires stakeholders at a variety of levels of the institution to champion its inclusion into the EMR. Given the encouraging results of this study, we are optimistic that the DST will eventually be incorporated in a way that all clinical providers can easily access and utilize the DST. Second, the DST is based upon a population PK model that was built using data from a single center (Primary Children's Hospital, Salt Lake City, UT). While the data from this center represents a large region in the Intermountain West, this region reflects a mostly Caucasian population. It is therefore possible that the PK model does not account for all covariates that significantly impact the disposition of tacrolimus in children. To address this limitation, we have partnered with other institutions (Seattle, Boston, and Texas Children's Hospitals) to test the current model with data from more heterogeneous populations. This work demonstrated the current model, with the additional inclusion of weight as a covariate on both volume and elimination rate, successfully predicted future concentrations at these external sites.^19^ Despite this success, we acknowledge a need to continually develop and finetune the underlying population PK model, for example, when an alternative azole antifungal other than fluconazole (or other CYP3A inhibitor) is used or when *CYP3A4/5* genotypes are collected prior to transplant. Participant genotypes were not available in the retrospective dataset used to construct the PK model the DST is based on, however, work is ongoing to incorporate genotype into the model and DST. Though these findings are outside the scope of the current manuscript, our work suggests a 2.7‐fold increase in tacrolimus dose requirement for individuals with the CYP3A5*1 allele relative to those with the CYP3A5*3/*3 genotype (data not shown), similar to prior reports in adult and pediatric heart transplant populations.^24^, ^25^, ^26^, ^50^ Next, the prospective study was compared to a historical cohort with a different (i.e., younger) age distribution. Older children are expected to more rapidly attain stable therapeutic concentrations relative to younger children, which may have benefited the success of the DST. A future randomized, controlled trial is needed to confirm the DST's utility.

A final limitation of the described DST is its performance in the setting of CRRT. While tacrolimus is primarily eliminated through hepatic metabolism, the DST utilizes creatinine clearance as a significant covariate required to predict the correct dose. Though the underlying physiology of this association is unclear, its importance is underscored by the DST's success in predicting the correct dose for a wide range of renal functions post‐transplant in study participants not treated with CRRT. CRRT plays a critical role in supporting patients with renal impairment, however, it is also well documented to impact the PK of some drugs due to the risk for drug binding to circuit components or elimination via filters within the circuit. Kishino et al reported that tacrolimus dose requirements were not altered in three adults co‐treated with continuous venovenous hemodiafiltration (CVVHD) following liver transplant.^51^ However, given the absence of additional literature evaluating the effect of CRRT on tacrolimus PK and the abundance of circuit materials and filters used in CRRT, the potential for an interaction between CRRT and tacrolimus PK is as yet unknown. The inability of the DST to guide dosing in CRRT patients supports the idea that the inclusion of renal function as a covariate within the DST likely serves as a surrogate measure of another physiologic parameter, such as altered blood flow, rather than its direct contribution to the elimination of tacrolimus. The current DST is therefore not recommended for use in patients receiving CRRT, until further studies are completed.

In summary, the described DST builds upon our prior work demonstrating the utility of a population PK model for guiding tacrolimus in pediatric heart transplant recipients. The study demonstrates that though the DST is independent from the PK modeling software NONMEM, the DST yields parameter estimates that match those found in NONMEM. Moreover, data from 15 participants indicate that use of the DST can hasten attainment of the correct tacrolimus dosing for pediatric heart transplant recipients. While more work is needed to implement this DST into the EMR and further demonstrate improved clinical outcomes, it is a promising advance toward personalized therapy for this population.

## Author Contributions

Jadon Wagstaff built and tested the decision support tool, and helped draft and edit the manuscript. Shaun S. Kumar was involved with model and decision support tool development and edited the manuscript. Kimberly M. Molina helped design the study, provided access to the studied patient population, and edited the manuscript. Joseph E. Rower constructed the population pharmacokinetic model, designed and secured funding for the prospective clinical trial, and wrote the manuscript.

## Conflicts of Interest

The authors have no financial or non‐financial competing interests to declare relevant to the described work.

## Funding

This research was supported by grant K01 HL148402 from the National Heart, Lung, and Blood Institute to Joseph E. Rower.

## Supporting information



Supporting Information

## Data Availability

Data for the study were collected from the Intermountain Healthcare Electronic Data Warehouse. Data that can be reasonably shared without compromising a participant's identity can be made available upon request. The decision support tool is publicly available on GitHub at https://github.com/jadonwagstaff/tacrodose_pht.
